# Magnitudes and Correlates of Human Immunodeficiency Virus, Hepatitis B Virus, and Syphilis among Pregnant Mothers Attending Antenatal Care in Addis Ababa, Ethiopia

**DOI:** 10.1155/2022/6156613

**Published:** 2022-02-16

**Authors:** Kassa Genetu, Kerebih Abere, Erdaw Tachbele

**Affiliations:** ^1^College of Health Sciences and Medicine, Dilla University, Dilla, Ethiopia; ^2^College of Health Sciences, Addis Ababa University, Addis Ababa, Ethiopia

## Abstract

**Background:**

Human immunodeficiency virus (HIV), hepatitis B virus (HBV), and syphilis are major sexually transmitted infections (STIs) among the general population in Ethiopia, which in turn result in a wide range of adverse pregnancy outcomes. Hence, it is important to determine the seroprevalence and risk factors of HIV, HBV, and syphilis infection among pregnant mothers attending antenatal care in Addis Ababa, Ethiopia.

**Method:**

A cross-sectional study was conducted among 286 pregnant women from February 1, 2021, to March 30, 2021, in four selected public hospitals in Addis Ababa. Sociodemographic, risky sociocultural, behavioral, and hospital-related factors were collected using an interview-administered questionnaire. Detection of anti-HIV antibodies, hepatitis B surface antigen (HBsAg), and rapid plasma reagin (RPR) for syphilis was conducted. A binary logistic regression analysis was used to determine predictors of STIs using SPSS version 25.

**Result:**

A total of 281 pregnant mothers with a mean age of 27.5 (SD 4.6) completed the study. Among the participants, the seroprevalence rates of HIV, HBV, and syphilis were 15 (5.3%), 9 (3.2%), and 5 (1.8%), respectively. Twenty six (9.3%) of the participants tested positive for any one of the STIs, and 3 (1.1%) of the women had HIV and syphilis coinfections. History of multiple sexual partners (AOR 3.42, 95% CI: 1.6-11.63) and STIs (AOR 3.7; 95% CI: 1.70-13.45) were significantly associated with HIV infection. Likewise, history of abortion (AOR 7.65, 95% CI: 1.17-49.74), tattooing (AOR 9.72, 95% CI: 1.41-66.73), and rape (AOR 9.72, 95% CI: 1.41-66.73) were significantly associated with hepatitis B virus infection. Husband history of multiple sexual partners (AOR 20.9, 95% CI: 1.8-241.8) was significantly associated with syphilis infection. The educational level of participants, history of tattooing (AOR 6.24, 95% CI: 1.79-21.7), and history of multiple sexual partners (AOR 5.15, 95% CI: 1.68-15.7) were independent predictors of infection with any one of the STIs.

**Conclusion:**

There is still a high burden of HIV, HBV, and syphilis among pregnant mothers in Ethiopia. History of multiple sexual partners, abortion, rape, and tattooing was a significant predictor of STIs.

## 1. Introduction

The global burden of HIV, HBV, and syphilis illnesses is a widespread problem among pregnant women, which in turn compromises the quality of life, reproductive health, and infant and child health [1, 2].

HIV remains to be the major public health problem of the world. At the end of 2020, there were 37.7 million people living with HIV with 680,000 HIV-related deaths and 1.5 million new infections [3]. Pregnant women account for approximately 48% of all new HIV infections and approximately half (52%) of all individuals living with HIV worldwide, and the infection is the leading cause of death among women of reproductive age [2, 4]. In Ethiopia, at the end of 2017, there were 650,000 people living with HIV with 17,200 AIDS-related deaths and 14,500 new infections [5]. In a meta-analysis, the pooled seroprevalence of HIV among pregnant women in Ethiopia was 5.74% [6].

HIV has adverse pregnancy outcomes on pregnant women like intrauterine growth restriction (IUGR) (20.5%), preterm birth (25.0%), and cesarean delivery (45.5%) [7, 8]. Besides, without any intervention, transmission rates of HIV from mother to child range from 15% to 45%. Similarly, HIV-infected and untreated children are at the highest risk of mortality within the first 3 months of life and up to 50% die within 2 years [3].

Globally, perinatal HBV spread accounts for an estimated 21% of HBV-related child deaths [9]. Africa has the biggest number of confirmed carriers after Asia and is considered a region of high endemicity [10]. It is assessed that between 70 and 95% of the adult population show evidence of past exposure to HBV infection and the prevalence of HBV disease among pregnant ladies ranges from 1.6% to13.8% [10]. Globally, 1.5 million people were newly infected with HBV in 2019, with 820,000 HBV infection-related deaths [3]. In Ethiopia, the pooled prevalence of HBV among the general population was 7.4% [11, 12], while it was 4.75% among the pregnant women [13, 14] that could be classified as WHO intermediate endemicity.

Syphilis, which is caused by *Treponema pallidum*, is still a global health problem despite the widely available methods of screenings and treatments. Globally, an estimated 0.8% of pregnant women and, in the African region, 2.9% of pregnant women are infected with syphilis [15]. In Ethiopia, the seroprevalence of syphilis among different subgroups of populations ranges from 1 to 10.9% [16]. The pooled seroprevalence of syphilis among pregnant women in Ethiopia was found to be 2.32% [17].

Untreated syphilis leads to an increment within the hazard of mother-to-child transmission (MTCT) of HIV. It has unfavorable pregnancy results, such as abortion, intrauterine fetal death, stillbirth, growth restriction, low birth weight, neonatal death, and congenital syphilis, due to untreated STIs [15, 18, 19]. Ethiopia is one of the three sub-Saharan countries with the highest numbers of adverse pregnancy outcomes caused by syphilis [19].

As recommended by the Global Health Sector Strategy on STI 2016–2021, diagnosing and treating these devastating etiologic agents at an early stage may result in preventing the spread of such infections and complications, for pregnant women as well as for their newborn infants [20]. Therefore, this study was designed to investigate the seroprevalence of HIV, HBV, and syphilis infections and their risk factors among pregnant women in Addis Ababa, Ethiopia.

## 2. Materials and Methods

### 2.1. Study Area, Design Period, and Population

A cross-sectional study design was conducted from February 1, 2021, to March 30, 2021, among 281 pregnant women in four randomly selected public hospitals, namely, Tikur Anbessa Specialized Hospital, Zewuditu Memorial Hospital, Ghandi Memorial Hospital, and St. Petros Hospital found in Addis Ababa, the capital city of Ethiopia. The city has 13 public hospitals, of which the four hospitals were selected by the lottery method. The study population was all pregnant women attending antenatal care at public hospitals in Addis Ababa during the study period. Women attending antenatal care who were willing to participate in the study and residing in Addis Ababa for at least 6 months were included in the study. Pregnant women who have severe health problems during the data collection period were excluded.

### 2.2. Sample Size Determination and Sampling Procedures

The sample size was determined using a single-population proportion formula by taking 6.5% the highest prevalence of HIV infection from the recent study in Dessie, Ethiopia [21], with an assumption of a 95% level of confidence and 3% margin of error. Accordingly, after adding the 10% nonresponse rate, a total of 286 pregnant women were included in the study.

The total sample size (*n* = 286) was allocated to each hospital proportionally based on the previous three-month average number of pregnant women attending antenatal care in each hospital. The study participants were chosen using the systematic random sampling method. The *k*^th^ interval of systematic sampling was determined by dividing the previous 3-month average number of mothers on antenatal care with the required sample size from each hospital.

### 2.3. Data Collection and Laboratory Diagnosis

An interview-administered questioner was used to collect data on the sociodemographic factors, risky sociocultural and behavioral practices, and hospital-related factors. All antenatal pregnant mothers receive routine laboratory diagnostic tests for HIV, HBV, and syphilis. Following the interview, 10 milliliters of venous blood sample was collected by sterile vein puncture procedure from each participant. The blood was centrifuged for 5 min at 300 revolutions per minute, and the serum sample was used for HIV, HBV, and syphilis screening.

HIV testing was done according to the national algorithm recommended by the Federal Ministry of Health of Ethiopia. Rapid HIV tests: HIV (1 + 2) rapid test strip Wantai (Beijing Wantai Biological Pharmacy Enterprise Co. Ltd., China) as the screening test, Uni-Gold HIV test® (Trinity Biotech Inc., Wicklow, Ireland) as a confirmatory test for positive samples, and VIKIA® HIV 1/2 (bioMérieux, SA, Marcy L'Etoile, France) as a tie-breaker test, were conducted in a series algorithm.

Sera were screened for syphilis using the rapid plasma reagin (RPR) test (Human GmbH-Wiesbaden, Germany), and the positives were retested using *Treponema pallidum* haemagglutination assay (TPHA) test (Guangzhou Wondfo Biotech Co. Ltd., Guangzhou, People's Republic of China). Pregnant women who tested positive for both RPR and TPHA were considered having *Treponema pallidum* infection.

All sera were tested for hepatitis B surface antigen (HBsAg) using rapid diagnostic tests that employ the principle of immunochromatography (SD Bioline, Yongin, Korea), which is believed to have an accuracy of above 98% and the best test to detect the current infection among pregnant women [22].

### 2.4. Data Quality Control and Analysis

For the data quality control, the questionnaire was checked and pretested before the actual data collection has been started. All the laboratory tests were performed following the manufacturers' protocol and quality in-built control assays. After checking for completeness and consistency of the collected information, the data was cleaned, coded, entered into Epi-Data 3.1, and transferred to Statistical Package for Social Sciences (SPSS) version 25 for analysis. The descriptive statistics (means, percentages, or frequency) was calculated, and the binary logistic regression analysis was used to see the relationship between dependent and independent variables. Variables having a *p* value of <0.20 in bivariate analysis were considered to have an association with the dependent variable and fitted into the multivariate analysis model. The strength of the association was measured using an odds ratio and interpreted by considering the 95% confidence interval, and *p* < 0.05 was a statistically significant risk factor in multivariate analysis.

### 2.5. Ethical Statement

Ethical approval was obtained from the Institutional Review Board of the School of Nursing and Midwifery, Addis Ababa University. Written consent was secured from study participants after a thorough explanation and understanding of the purpose of the study. Confidentiality and privacy of participants were maintained throughout the study process.

## 3. Results

### 3.1. Sociodemographic and Some Obstetric Characteristics of Study Participants

The study approached a total of 286 pregnant women, of which 281 participants completed the study, with a 98.2% response rate. The mean age of the participants was 27.5 where majority (199) (70.8%) of them were between 19 and 29 years old. Most of the study participants were married (256, 91.1%), and 116 (41.3%) attend secondary school. Among the study participants, 116 (41.3%) were government employees, 161 (57.3%) of them were multiparous, and 120 (42.7%) of them were multigravidas (Table 1).

### 3.2. Seroprevalence of HIV, HBV, and Syphilis Infections among the Study Participants

HIV, HBV, and syphilis were found to be prevalent in 15 (5.3%), 9 (3.2%), and 5 (1.8%) of the study participants, respectively, out of which 3 (1.1%) had HIV/syphilis coinfection. There were no coinfections with HIV/HBV, HBV/syphilis, or HIV/HBV/syphilis. Among the 281 pregnant women, 26 (9.3%) were found to be infected with any one of the three STIs.

### 3.3. Risky Hospital-Related, Sociocultural, and Behavioral Factors among Pregnant Women

Among the study participants, 29.9% of them had a history of hospital admission, 29.2% had history of operation for a surgical procedure, and 19.2% of them had a history of blood transfusion. More than 38% had a history of sexually transmitted infections, 65.1% have a history of premarital sex, majority (94.7%) of them had ear piercing, and 35.9% of them had history of multiple sexual partners (Table 2).

### 3.4. Seroprevalence and Associated Risk Factors of HIV, HBV, and Syphilis Infections among the Study Participants

To determine the relationship between each independent variable and HIV, HBV, and syphilis serostatus, binary logistic regression analysis was conducted. The independent variables with a *p* value of ≤ 0.20 were fitted into the multivariate logistic regression model to identify independent predictors of HIV, HBV, and syphilis infection (Tables 3–5).

### 3.5. Seroprevalence of HIV and Associated Factors

The total prevalence of HIV was 5.3% (95% CI: 2.8-8.2). In bivariate logistic regression, women's history of multiple sexual partners, history of husband multiple sexual partners, history of women STIs, husband history of STIs, and history of surgical operation were correlated with the seroprevalence of HIV infection (*p* ≤ 0.05). However, in multivariate analysis, history of women's STIs and history of husband sexual partner remained independent predictors of HIV infection. Women having a history of STIs were 3.79 times (AOR 3.79; 95% CI: 1.70-13.45) more likely to be infected by HIV than those who did not have STIs. Likewise, pregnant women whose husbands had a history of multiple sexual partners had more than threefold (AOR 3.42; 95% CI: 1.60-11.63) chance of acquiring HIV infection compared to their counterparts (Table 3).

### 3.6. Seroprevalence of Hepatitis B Virus (HBV) and Associated Factors among the Study Participants

The seroprevalence of HBV was 3.2% (95% CI: 1.1-5.3). In bivariate logistic regression, history of abortion, dental procedure, hospital admission, and tattooing were associated with seroprevalence of HBV infection (*p* ≤ 0.05). However, in multivariate analysis, history of tattooing, abortion, and rape were found to be independent predictors of HBV infection. Women with a history of tattooing were twelve times (AOR 11.88, 95% CI: 2.17-16.02) more likely to be infected with HBV compared to those who did not have tattooing. Similarly, having histories of abortion (AOR 7.65, 95% CI: 1.17-9.74) and rape (AOR 9.72, 95% CI: 1.41-16.73) was more than 7 and 9 times more likely to be infected by HBV infection than those who did not have these histories (Table 4).

### 3.7. Seroprevalence of Syphilis and Associated Factors among the Study Participants

The overall prevalence of syphilis was 1.8% (95% CI: 0.4-3.8). In multivariate analysis, women whose partners have history of multiple sexual partners were 20 times (AOR 20, 95% CI: 1.8-41.8) more likely to be infected by syphilis than their counterparts.

### 3.8. Seroprevalence and Associated Factors for Any of the 3 STIs (HIV, HBV, and Syphilis)

Out of the 281 pregnant women, 26 was found to be reactive for any of the 3 STIs with an overall prevalence of 9.3% (95% CI: 6.0-12.8). In bivariate analysis, the educational level of respondents, history of dental procedure, history of blood transfusion, history of abortion, history of tattooing, history of multiple sexual partners, and history of husband's sexual partner were associated with the overall prevalence of STIs. However, in multivariate analysis, the educational level of respondents, history of tattooing, and history of multiple sexual partner were significantly associated with the overall occurrence of sexually transmitted infections. Accordingly, women with an educational level of secondary school were 89% times (AOR 0.11, 95% CI: 0.03-0.41) less likely to be infected with STIs than those who did not have formal education. Similarly, pregnant women who have an educational level of college and above were 81% (AOR 0.19, 95% CI: 0.04-0.77) less likely to be infected by STIs. Likewise, women having a history of tattooing were 6 times (AOR 6.24; 95% CI: 1.79-21.70) more likely to be infected by STIs than their counterparts. Furthermore, women with a history of multiple sexual partners had a fivefold chance of getting any one of the STIs than their counterparts (AOR 5.15, 95% CI:1.68-15.7) (Table 5).

## 4. Discussion

This study pursued to estimate the prevalence of the 3 STIs and identify the risk factors of infection of HIV, HBV, and syphilis among pregnant women attending antenatal care at public hospitals in Addis Ababa, Ethiopia.

In the present study, the seroprevalence of HIV among pregnant women was 5.3%, which is in line with studies done in Cameroon (5.0%) [23], sub-Saharan African countries (5.3%) [24], and Ethiopia (5.74%) [6]. This finding was lower than the previous studies done in China (6.6%) [25], Cameroon (13.1%) [26], Gondar (11.2%) [27], and Dessie (6.5%) [21]. However, it is higher than a study done in Addis Ababa (4.8%) [6], Gondar (4.1%) [28], Sudan (1.13%) [29], Guatemala (0.21%) [30], and Angola (2.6%) [31]. The variations might be the reflections of differences in sociocultural practices, accessibility to the health care system, risky sexual and behavioral practices, and awareness of HIV infection. This study indicates that HIV is still an important public health concern among pregnant women in Addis Ababa and suggests that there is a need to strengthen intervention efforts including health education towards voluntary counseling and testing, modes of transmission, prevention, and mother-to-child transmission of HIV infection. The present study demonstrated that study participants with a history of other STIs were 3.79 times more likely to be infected with HIV than those without previous history of STIs. This finding was supported by a research done in Dessie, Ethiopia [21], and Addis Ababa [6] in which the history of STIs was associated with HIV infection. This might be because STIs create ulcer on the skin and mucous membrane which in turn facilitates the acquisition of HIV infection. Pregnant women having a husband with a history of multiple sexual partners have 3.42 times more chance to acquire HIV infection compared to their counterparts. Studies conducted in Dessie, Ethiopia [21], Addis Ababa [6], Uganda [32], Cameroon [26], and Southwest Nigeria [33] showed that the habit of multiple sexual practices was significantly associated with the acquisition of HIV infection. This may be because husbands of pregnant women have risky sexual behavior that facilitates the acquisition of HIV both for them and for the women.

In this study, the overall prevalence of hepatitis B infection was 3.2%, which can be graded as intermediate endemicity according to WHO criteria [11]. This result is consistent with a study conducted in Kenya (3.8%) [34], Tanzania (3.9%) [35], Dawro (3.5%) [36], and Bahir Dar, Ethiopia (3.8%) [37]. Conversely, this finding was lower than studies done in Kano, Nigeria (7.9%) [33], Ghana (9.5%) [38], Angola (7.5%) [31], Solomon Islands (13.8%) [39], Deder (6.9%) [40], Arbaminch (4.3%) [41], Dessie (4.7%), Yirgalem (7.2%) [42], and southern Ethiopia (7.8%) [43]. However, it is higher than studies reported in Egypt (1.6%) [44] and Sudan (2.93%) [29]. The differences observed in the prevalence of hepatitis B virus prevalence across a different country might be due to differences in sociodemographic characteristics, risky sexual and behavioral practices, sociocultural environment, and traditional operations where the study participants are residing.

According to the current study, women who had history of abortion were 7.65 times more likely to be infected with HBV than women who had never had an abortion. Similarly, research conducted in Nigeria [33] and Deder [40] showed that women history of abortion was substantially linked with HBV infection. Participants in the study who had a history of rape were found to be 9.72 times more infected than their peers. The present study showed that pregnant women with a history of tattooing were almost twelve times more infected than those who did not have this phenomenon. This could be because tattooing was done in a conventional fashion, with the risk of using common sharp tools, which would facilitate the spread of hepatitis B virus infection.

The total frequency of syphilis among pregnant women was 1.8 percent in this study. This finding was consistent with research done in Bahir Dar (2.9%) [45]. Yet, the prevalence of syphilis is higher than 0.6% of Dessie, Ethiopia [21]. Compared to other countries, the observed 1.8% seroprevalence of syphilis is lower than the 5.7% prevalence in Cameroon [23], 2.9% in sub-Saharan African countries [15], 3.2% in the east African region [15], and 3.8% in South African regions [15]. The lower prevalence of syphilis among pregnant women might be attributed to variations in sexual and behavioral practices, difference in access to treatment of STIs, the difference in cultural practice, and the increase in syphilis screening at every antenatal care visit.

The present study showed that pregnant women whose husbands have a history of multiple sexual partners were significant risk factors for the acquisition of syphilis infection. Women whose husbands/partners have a history of having a new sex partner in the last 6 months were 20.97 times more infected with syphilis infection. Similarly, studies in the Ekiti state, Nigeria [46], and Bahir Dar [45] showed that a history of multiple sexual partners of women had an association with syphilis infection.

In this study, pregnant women having an educational level of secondary and above the secondary were less likely to be infected with sexually transmitted infections than those who did not have formal education. This may be because respondents might have an awareness regarding sexually transmitted infections, mother-to-child transmission, prevention, and complications of STIs in different youth and schools' anti-AIDS and reproductive health clubs.

## 5. Conclusion and Recommendation

In this study, the history of multiple sexual partners, history of tattooing, history of abortion, history of sexually transmitted infections, and history of rape were significantly associated with HIV, hepatitis B virus, and syphilis infection. There is still a high burden of HIV, HBV, and syphilis among antenatal mothers in Ethiopia. Therefore, it is important to strengthen the existing antenatal care services and screen STIs among all pregnant women at every antenatal care visit.

## Figures and Tables

**Table 1:** Sociodemographic and some obstetric characteristics of pregnant mothers visiting antenatal clinics at selected hospitals in Addis Ababa, February, 2021 (*n* = 281).

Variables	Categories	Frequency	Percent
Age	19–29	199	70.8
	30–39	77	27.4
	40–49	5	1.8
Marital status	Single	25	8.9
	Married	256	91.1
Educational status	No formal education	34	12.1
	Primary school	55	19.6
	Secondary school	116	41.3
	College and above	76	27
Occupation	Employed	116	41.3
	Unemployed	102	36.3
	Farmer	5	1.8
	Private	58	20.6
Gravidity	Primigravida	24	8.5
	Multigravida	257	91.5
Parity	Primiparous	121	43.1
	Multiparous	160	56.9

**Table 2:** Risky characteristics and distribution of the three STIs among pregnant women attending antenatal care at selected hospitals in Addis Ababa, February to March, 2021 (*n* = 281).

Variables	Categories	Frequency, *n* (%)	HIV, *n* (%)	HBV, *n* (%)	Syphilis, *n* (%)	Any STI, *n* (%)
History of hospital admission	No	197 (70.1)	9 (3.2)	3 (1.0)	4 (1.4)	14 (5.0)
	Yes	84 (29.9)	6 (2.1)	6 (2.1)	1 (0.4)	12 (4.8)
History of dental procedure	No	220 (78.3)	9 (3.2)	3 (1.0)	3 (1.0)	14 (5.0)
	Yes	61 (21.7)	6 (2.1)	6 (2.1)	2 (0.7)	12 (4.8)
History of operation for a surgical problem	No	197 (70.1)	7 (2.5)	9 (3.2)	3 (1.0)	18 (6.4)
	Yes	84 (29.9)	8 (2.8)	0 (0.0)	2 (0.7)	8 (2.8)
History of blood transfusion	No	227 (80.8)	10 (3.6)	5 (1.8)	3 (1.0)	17 (6.0)
	Yes	54 (19.2)	5 (1.8)	4 (1.4)	2 (0.7)	9 (3.2)
History of women STIs	No	175 (62.3)	4 (1.4)	6 (2.1)	2 (0.7)	12 (4.8)
	Yes	108 (38.4)	11 (3.9)	5 (1.8)	3 (1.0)	14 (5.0)
History of partner STIs	No	187 (66.5)	6 (2.1)	7 (2.5)	1 (0.4)	13 (4.6)
	Yes	94 (33.5)	9 (3.2)	2 (0.7)	4 (1.4)	13 (4.6)
History of abortion	No	235 (83.6)	12 (4.3)	3 (1.0)	4 (1.4)	17 (6.0)
	Yes	46 (16.4)	3 (1.0)	6 (2.1)	1 (0.4)	9 (3.2)
History of tattooing	No	244 (86.8)	12 (4.3)	3 (1.0)	4 (1.4)	16 (5.7)
	Yes	37 (13.2)	3 (1.0)	6 (2.1)	1 (0.4)	10 (3.6)
History of ear piercing	No	15 (5.3)	1 (0.4)	1 (0.4)	0 (0.0)	2 (0.7)
	Yes	266 (94.7)	14 (5.0)	8 (2.8)	5 (1.8)	24 (8.5)
History of nose piercing	No	267 (95)	14 (5.0)	9 (3.2)	5 (1.8)	25 (8.9)
	Yes	14 (5.0)	1 (0.4)	0 (0.0)	0 (0.0)	1 (0.4)
History of premarital sex	No	98 (34.9)	7 (2.5)	3 (1.0)	5 (1.8)	12 (4.8)
	Yes	183 (65.1)	8 (2.8)	6 (2.1)	0 (0.0)	14 (5.0)
Share sharp instruments with others	No	172 (61.2)	9 (3.2)	5 (1.8)	0 (0.0)	14 (5.0)
	Yes	109 (38.8)	6 (2.1)	4 (1.4)	5 (1.8)	12 (4.8)
History of rape	No	212 (75.4)	11 (3.9)	5 (1.8)	2 (0.7)	17 (6.0)
	Yes	69 (24.6)	4 (1.4)	4 (1.4)	3 (1.0)	9 (3.2)
History of women with multiple sexual partners	No	180 (64.1)	5 (1.8)	6 (2.1)	1 (0.4)	11 (3.9)
	Yes	101 (35.9)	10 (3.6)	3 (1.0)	4 (1.4)	15 (5.3)
History of husband with multiple sexual partners	No	220 (78.3)	8 (2.8)	7 (2.5)	1 (0.4)	16 (5.7)
	Yes	61 (21.7)	7 (2.5)	2 (0.7)	4 (1.4)	10 (3.6)

**Table 3:** Associated factors of HIV infection among pregnant women attending antenatal care at selected public hospitals in Addis Ababa, February to March, 2021 (*n* = 281).

Variables	Category	Frequency	HIV serostatus		Bivariate analysis	Multivariate analysis	
			No, *n* (%)	Yes, *n* (%)	COR (95% CI)	AOR (95% CI)	*p* value
Marital status	Single	25	22 (7.8)	3 (1.0)	1	1	**—**
	Married	256	244 (87)	12 (4.0)	0.361 (0.095-1.37)	0.56 (0.10-3.18)	0.51
Dental procedure	No	220	211 (75)	9 (3.2)	1	1	—
	Yes	61	55 (19.5)	6 (2.1)	2.55 (0.873-7.492)	1.53 (0.42-5.52)	0.51
Hx of surgical procedure	No	197	190 (68)	7 (2.4)	1	1	
	Yes	84	76 (27)	8 (2.8)	2.85 (1.1-8.154)	2.83 (0.88-9.10)	0.08
Hx of blood transfusion	No	227	217 (77)	10 (4.0)	1	1	**—**
	Yes	54	49 (17.4)	5 (1.7)	2.21 (0.72-6.76)	2.11 (0.54-8.25)	0.28
History of woman STIs	No	173	169 (60)	4 (1.4)	1	1	**—**
	Yes	108	97 (34.5)	11 (4)	4.79 (1.48-15.45)^∗^	3.7 (1.70-13.45)	0.03^∗∗^
History of husband STIs	No	187	181 (64)	6 (2.1)	1	1	**—**
	Yes	94	85 (30.2)	9 (3.2)	3.19 (1.10-9.26)^∗^	2.07 (0.63-6.71)	0.22
Hx of husband with multiple sexual partners	No	220	212 (75)	8 (2.8)	1	1	**—**
	Yes	61	54 (19.2)	7 (2.4)	3.43 (1.19-9.89)^∗^	3.42 (1.6-11.63)	0.04^∗∗^
Hx of woman with multiple sexual partners	No	180	175 (62)	5 (1.7)	1	1	**—**
	Yes	101	91 (32.3)	10 (4.0)	3.84 (1.27-11.58)^∗^	2.20 (0.66-7.31)	0.19

^∗^In a bivariate analysis, *p* ≤ 0.05 is statistically significant. ^∗∗^In multivariate analysis, *p* ≤ 0.05 is statistically significant; 1: reference category; STIs: sexually transmitted infections; Hx: history.

**Table 4:** Associated factors of hepatitis B infection among pregnant women attending antenatal care at selected public hospitals in Addis Ababa, February to March, 2021 (*n* = 281).

Variables	Category	Frequency	HBV serostatus		Bivariate analysis	Multivariate analysis	
			No, *n* (%)	Yes, *n* (%)	COR (95% CI)	AOR (95% CI)	*p* value
History of dental procedure	No	220	217 (98.6)	3 (1.4)	1	1	—
	Yes	61	55 (90.2)	6 (9.8)	7.89 (1.9-32.55)^∗^	4.86 (0.79-29.63)	0.08
History of blood transfusion	No	227	222 (97.8)	5 (2.2)	1	1	**—**
	Yes	54	50 (92.6)	4 (7.4)	3.55 (0.92-13.7)^∗^	1.98 (0.32-12.10)	0.45
History of hospital admission	No	197	194 (98.5)	3 (1.5)	1	1	**—**
	Yes	84	78 (92.9)	6 (7.1)	4.97 (1.2-20.38)^∗^	1.90 (0.34-10.51)	0.46
History of abortion	No	235	232 (98.7)	3 (1.3)	1	1	**—**
	Yes	46	40 (87.0)	6 (13.0)	11.6 (2.7-48.27)^∗^	7.65 (1.17-19.74)	0.03^∗∗^
History of rape	No	212	207 (97.6)	5 (2.4)	1	1	**—**
	Yes	69	65 (94.2)	4 (5.8)	2.54 (0.66-9.76)	9.72 (1.41-16.73)	0.02^∗∗^
History of tattooing	No	244	241 (98.8)	3 (1.2)	1	1	**—**
	Yes	37	31 (83.8)	6 (16.2)	15.54 (3.7-65.3)^∗^	11.8 (2.17-16.02)	0.004^∗∗^

^∗^In a bivariate analysis, *p* ≤ 0.05 is statistically significant. ^∗∗^In multivariate analysis, *p* ≤ 0.05 is statistically significant; 1: reference category.

**Table 5:** Associated factors of any one of the STIs among pregnant women attending antenatal care at selected public hospitals in Addis Ababa, February to March, 2021 (*n* = 281).

Variables	Category	Frequency	Any STI serostatus		Bivariate analysis	Multivariate analysis	
			No, *n* (%)	Yes, *n* (%)	COR (95% CI)	AOR (95% CI)	*p* value
Gravidity	Primigravida	24	21 (87.5)	3 (12.5)	1	1	—
	Multigravida	257	234 (91.1)	23 (8.9)	0.69 (0.19-2.48)		
Parity	Primiparous	121	106 (87.6)	15 (12.4)	1	1	—
	Multiparous	160	149 (93.1)	11 (6.9)	0.52 (0.23-1.18)	0.55 (0.17-1.76)	0.32
Educational level	No formal education	34	22 (64.7)	12 (35.3)	1	1	—
	Primary school	55	55 (100)	0 (0.0)			
	Secondary school	116	109 (94)	7 (6)	0.11 (0.04-0.33)^∗^	0.11 (0.03-0.41)	0.004^∗∗^
	College and above	76	69 (90.8)	7 (9.2)	0.18 (0.05-0.53)^∗^	0.19 (0.04-0.77)	0.020^∗∗^
Hx of hospital admission	No	197	183 (92.9)	14 (7.1)	1	1	—
	Yes	84	72 (85.7)	12 (14.3)	2.18 (0.96-4.93)	1.59 (0.50-5.11)	0.43
History of dental procedure	No	220	206 (93.6)	14 (6.4)	1	1	—
	Yes	61	49 (80.3)	12 (19.7)	3.60 (1.57-8.28)^∗^	2.31 (0.70-7.54)	0.16
History of blood transfusion	No	227	210 (92.5)	17 (7.5)	1	1	—
	Yes	54	45 (83.3)	9 (16.7)	2.47 (1.04-5.90)^∗^	2.76 (0.80-9.55)	0.11
History of woman STIs	No	173	161 (93.1)	12 (6.9)	1	1	—
	Yes	108	94 (87)	14 (7)	2.0 (0.89-4.50)	1.67 (0.54-5.11)	0.37
History of husband STIs	No	187	174 (93)	13 (7)	1	1	—
	Yes	94	81 (86.2)	13 (13.8)	2.15 (0.95-4.84)	2.86 (0.97-8.40)	0.06
History of abortion	No	235	218 (92.8)	17 (7.2)	1	1	—
	Yes	46	37 (80.4)	9 (19.6)	3.11 (1.30-7.52)^∗^	0.75 (0.16-3.39)	0.71
History of tattooing	No	244	228 (93.4)	16 (6.6)	1	1	—
	Yes	37	27 (73)	10 (27)	5.28 (2.18-12.79)^∗∗∗^	6.24 (1.79-21.7)	0.004^∗∗^
History of premarital sex	No	98	86 (87.8)	12 (12.2)	1	1	—
	Yes	183	169 (92.3)	14 (7.7)	0.59 (0.26-1.34)	0.34 (0.10-1.16)	0.08
Hx of multiple sexual partners	No	180	169 (93.9)	11 (6.1)	1	1	—
	Yes	101	86 (85.1)	15 (14.9)	2.68 (1.18-6.08)^∗^	5.15 (1.68-15.7)	0.004^∗∗^
History of rape	No	212	195 (92)	17 (8)	1	1	—
	Yes	69	60 (87)	9 (13)	1.72 (0.72-4.06)	2.69 (0.80-8.98)	0.11
Hx of husband with multiple sexual partners	No	220	204 (92.7)	16 (7.3)	1	1	—
	Yes	61	51 (83.3)	10 (16.4)	2.50 (1.07-5.83)^∗^	1.92 (0.59-6.26)	0.28

^∗^In a bivariate analysis, *p* ≤ 0.05 is statistically significant. ^∗∗^In multivariate analysis, *p* ≤ 0.05 is statistically significant; 1: reference category. Sexual history of the women's husband.

## Data Availability

The data used to support the findings of this study are available from the corresponding author upon request. The source of this article is a thesis found in Addis Ababa University Institutional repository at http://etd.aau.edu.et/handle/123456789/28585 [47].

## Abbreviations

ANC: Antenatal care
AOR: Adjusted odds ratio
CI: Confidence interval
EDHS: Ethiopia demographic and health survey
HbsAg: Hepatitis B surface antigen
HBV: Hepatitis B virus
HIV: Human immunodeficiency virus
IRB: Institutional review board
MTCT: Mother-to-child transmission
OR: Odds ratio
SPSS: Statistical package social sciences
STIs: Sexually transmitted infections
WHO: World Health Organization.
